# A case of thymoma in myasthenia gravis: Successful outcome after thymectomy^☆^

**DOI:** 10.1016/j.ijscr.2019.10.069

**Published:** 2019-11-05

**Authors:** S. Dahal, N. Bhandari, P. Dhakal, R.M. Karmacharya, A.K. Singh, S.M. Tuladhar, M. Devbhandari

**Affiliations:** Kathmandu University School of Medical Sciences, Department of Surgery, Dhulikhel, 3, Nepal

**Keywords:** Case report, Myasthenia gravis, Nepal, Thymectomy, Thymoma

## Abstract

**Introduction:**

Thymic abnormalities occur as hyperplasia and thymoma. Myasthenia gravis is commonly present in thymoma. Thymectomy possesses risk due to anatomical proximity with vital thoracic structures and myasthenia crisis.

**Presentation of case:**

Forty five years female with complaints of difficulty swallowing and weakness of upper limb muscles upon investigation showed mass in mediastinum and antibody test for myasthenia gravis positive. Medical management was done for a month followed by thymectomy. There were no intra and postoperative complications. Medical management was stopped one month after surgery and she is symptom free.

**Discussion:**

Thymectomy is the standard of care where median sternotomy is the mainstay approach to surgery. Various other surgical approaches and complications revolving around surgery has been discussed.

**Conclusion:**

Surgical removal of thymoma cured myasthenia gravis in our case. We focused on proper preoperative optimization of myasthenia gravis symptoms before thymectomy.

## Highlights

• Thymoma can present with Myasthenia Gravis.
• Thymectomy improves outcome of Myasthenia gravis in patients with Thymoma.
• Complications in thymectomy can be prevented by proper pre- operative optimization and meticulous dissection during surgery.

## 1 Introduction

Thymomas are rare but common neoplasm of mediastinum in adults [1]. Thymic abnormalities are prevalent in form of hyperplasia in 60%–70% and thymoma in 10%–15%; myasthenia gravis is present in 15–20% of thymoma patients [2,3]. Myasthenia gravis is a disease of the neuromuscular junction which causes progressive weakness of muscles [2]. Indication of thymectomy for all cases of myasthenia gravis has been a topic of debate but thymectomy is indicated in all cases with thymomas no matter the stage of myasthenia gravis [2]. Thymectomy is a challenging procedure not only because of its close relation to heart, great vessels and lungs requiring cardiac risking thoracic surgical emergencies but also because this procedure poses significant challenge for anesthetic team during intraoperative and postoperative period. This is due to the possibility of mechanical compression of airway and myasthenia crisis during the procedure [4]. There are very few cases reported for thymoma in myasthenia gravis patient in our context. The case is unique because the patient did not present with symptoms early despite the large tumor size. Myasthenia Gravis symptoms successfully resolved after the surgical treatment and no medical therapy was required. Hence we report this case of thymoma in myasthenia gravis. This work is reported in accordance with SCARE Criteria [5].

## 2 Presentation of case

**Fig. 1:**
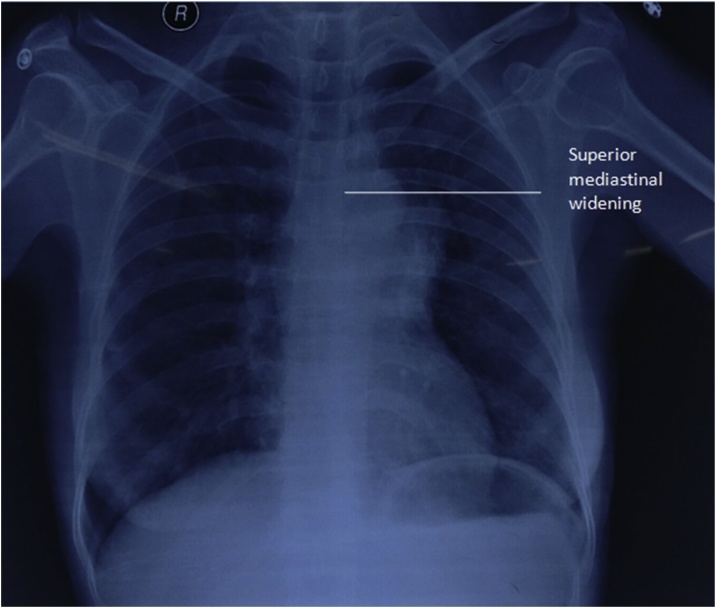
Chest X-ray showing mass in superior mediastinum.

**Fig. 2:**
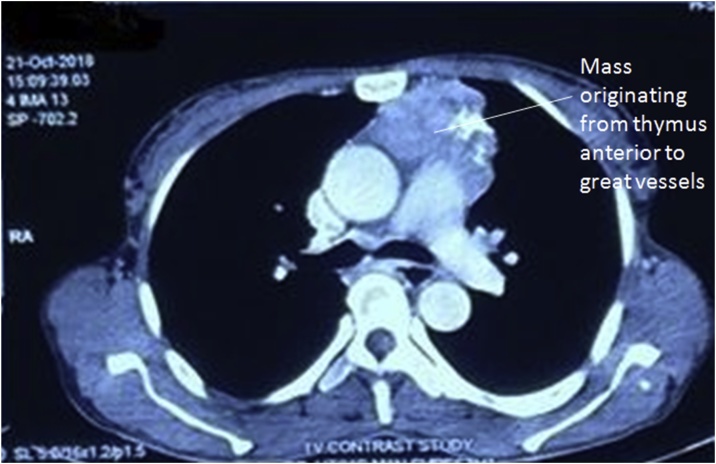
CT showing anterior mediastinal mass.

**Fig. 3:**
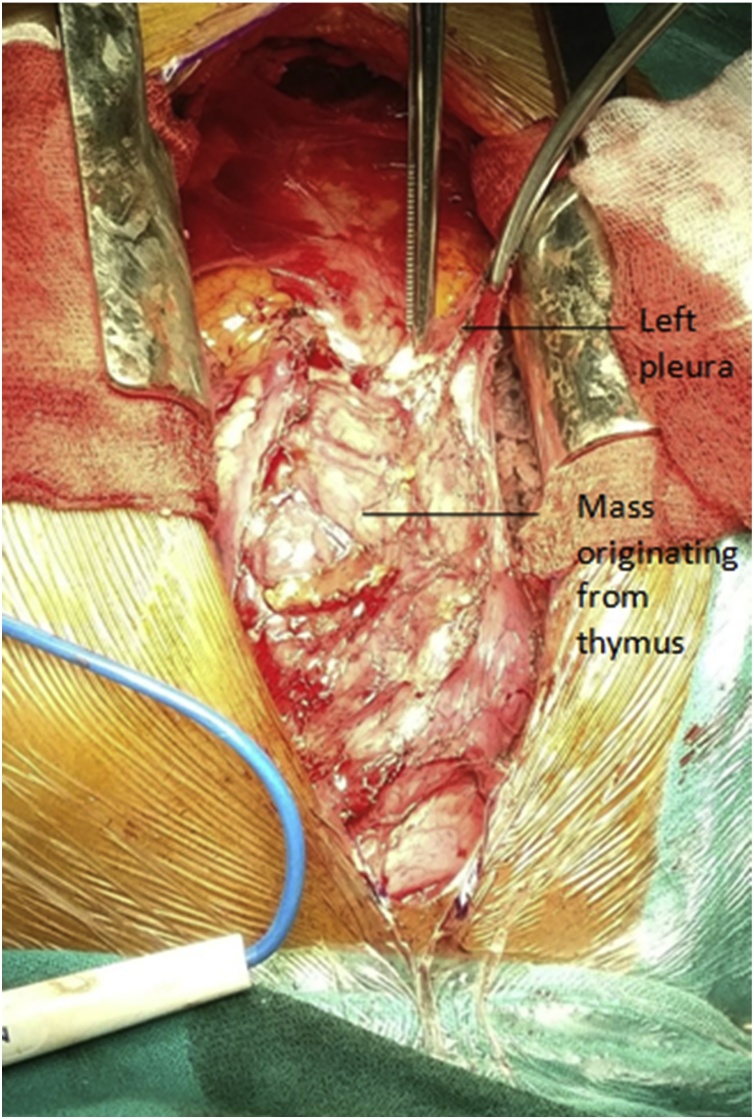
Intraoperative picture showing thymoma being separated from left pleura.

Forty five years female from hilly region of Nepal presented to our Hospital, a community based hospital, with difficulty of swallowing for seven months initially for solid foods which gradually progressed to liquid foods. She had difficulty breathing since two months. There was weakness of upper limb muscles, more pronounced in the evening. There was no decrease in appetite, waterbrash, weight loss or cough. Chest X-ray was suggestive of mediastinal widening (Fig. 1). Contrast enhanced computed tomography was done which suggested mediastinal mass originating from thymus with size of 12 × 12 cm (Fig. 2). Antibody tests for myasthenia gravis were positive (8.67 nmol/L). She was medically managed for a month with oral pyridostigmine 60 mg per day and definitive surgery was done. After sternotomy, the mass exposed was 12 × 13 cm in anterior mediastinum originating from thymus and encasing the left phrenic nerve, abutting aorta and pericardium (Fig. 3). Tumor was dissected free from the innominate vein and left superior pulmonary vein. Single lymph node of size 3 × 2 cm was also dissected from the origin of left internal mammary artery. Lung surface and the pericardium were free of tumor. Postoperative period was uneventful and she was discharged on the seventh postoperative period with need of intensive care unit for the first two postoperative days. During follow up the patient took pyridostigmine for a month, after which medication was stopped considering the absence of myasthenia gravis symptoms. Recurrence of thymoma was not evident until 6 months. The thymoma specimen was 8 × 8 × 3 cm with multiple nodular areas. Largest nodule was 3 × 3 cm with cystic areas. Histopathology showed Thymoma of World Health Organization(WHO) Stage B2 with reactive changes in lymph nodes. Following surgery and alleviation of symptoms the patient denied any form of complementary therapy. However, the patient has been called for 3 monthly follow up.

## 3 Discussion

Thymoma may present with a range of clinical features from asymptomatic detected incidentally upon investigations, presenting with local symptoms to features of myasthenia gravis or other neoplastic processes. One-third of the patient present with space occupying features while one-third to two-thirds have complaints of autoimmune condition, most commonly myasthenia gravis present in 15 to 20 percent of cases [6]. Management for thymoma is done with multimodality diagnosis and treatment strategies, surgery i.e Thymectomy is the standard of care [6,7]. Open surgery with median sternotomy is the mainstay approach to Thymectomy [8]. Minimally invasive methods have been tried displaying similar outcomes to open surgery in selected cases but has higher risk of incomplete thymectomy [8]. A recent systematic review and meta-analysis shows superiority of Robotic thymectomy over open and video-assisted thoracoscopic surgery (VATS) but VATS has not been found superior to open thymectomy [9]. Thymectomy poses surgical difficulty due to close proximity with vital structures like pericardium, great vessels, phrenic nerve and lungs. Hence meticulous dissection and minimal use of electrocautery near phrenic nerve area with adequate hemostasis is necessary [10]. For better prognosis after thymectomy, en-bloc resection of the entire thymus with surrounding tissue in pre-vascular plane of anterior mediastinum between phrenic nerves is done [11]. Myasthenia crisis is the most common cause of operative mortality; other causes are airway compromise, infection and nerve palsies [2,12]. Myasthenia crisis can be reduced by preoperative neurological assessment, CT scan and pulmonary function test; operating during stable phase of myasthenia gravis and, low use of steroids and neuromuscular blockade during anesthesia [13]. Pyridostigmine is used for a month before surgery to stabilize myasthenia gravis [2]. Postoperative challenges occur during recovery and extubation [2,14]. The management of myasthenia crisis involves securing the airway and maintaining of respiratory function [13]. Since the tumor is rare, proper staging system was not used regularly before 1981 [15,16]. Commonly used staging system for this condition is WHO staging according to which Stage B2 includes tumor in which there is Increased density of dispersed or clustered epithelial cells (≥3 adjacent cells) with moderately dense immature lymphocyte population [17]. Transsternal thymectomy is commonly used approach for thymectomy which has advantages like adequate exposure of the tumor along with adjacent vital organs [18]. Chest infection, phrenic nerve palsy, respiratory failure are some common documented complications after thymectomy [18]. Locally advanced tumors require adjuvant chemoradiation therapy [6].

## 4 Conclusion

Surgical removal of thymoma cured the problem of myasthenia gravis in our case. We focused on proper preoperative optimization of myasthenia gravis symptoms before thymectomy. During surgery, care was taken to prevent damage to adjacent vital organs. Vigilance to note myasthenia crisis and proper management in such scenario is vital for successful results.

## Sources of funding

There is no external funding provided to the study.

## Ethical approval

Since this is a case report, ethical approval was not required from Institutional Review Board.

## Consent

Written informed consent was obtained from the patient for publication of this case report and accompanying images. A copy of the written consent is available for review by the Editor-in-Chief of this journal on request.

## Author contribution

Study Concept: S Dahal, N Bhandari, P Dhakal, RM Karmacharya.

Data Collection: RM Karmacharya.

Writing: S Dahal, N Bhandari, P Dhakal, RM Karmacharya, AK Singh.

Revising and critical review: M Devbhandari, SM Tuladhar.

## Registration of research studies

This is a case report so registration was not required.

## Guarantor

All the authors are the guarantor of the study.

## Provenance and peer review

Not commissioned or externally peer reviewed.

## Declaration of Competing Interest

There are no conflict of interests.
